# The B-cell compartment in antibody-deficient infants and young children – developing common variable immunodeficiency or transient immune maturation?

**DOI:** 10.1186/s13052-016-0279-y

**Published:** 2016-07-26

**Authors:** Aleksandra Szczawinska-Poplonyk, Katarzyna Tapolska-Jozwiak, Husam Samara

**Affiliations:** 1 Department of Pediatric Pneumonology, Allergology and Clinical Immunology, Karol Marcinkowski University of Medical Sciences, Szpitalna Street 27/33, 60-572 Poznan, Poland; 2 Department of Immunology, Karol Marcinkowski University of Medical Sciences, Rokietnicka 5D, 60-806 Poznan, Poland

**Keywords:** Hypogammaglobulinemia, Immunophenotype, B lymph cells, Children

## Abstract

**Background:**

Hypogammaglobulinemia in early childhood is a common feature characterized by distinct intrinsic and extrinsic factors leading to disturbed peripheral blood lymphocyte homeostasis. Detailed flow cytometric immunophenotyping of the peripheral blood B cell compartment is an informative tool for delineating disturbed generation of B cell subpopulations crucial for the diagnosis of hypogammaglobulinemia in young children.

**Methods:**

We analyzed by flow cytometry the proportions and absolute values of total, naïve, memory - non-switched and switched, transitional and immature B lymph cells as well as plasmablasts in the peripheral blood of 50 hypogammaglobulinemic children aged from 3 to 50 months.

**Results:**

Beyond physiological, age-related changes within the B cell pool, a proportion of children manifested defective differentiation into switched memory and accumulation of CD21lo immature B cells.

**Conclusions:**

Dynamic shifts within B cell subpopulations of the immature immune system being most prominent during the first two years of life contribute to the age-related developmental abnormalities of the B cell compartment. Therefore, a reliable diagnosis of common variable immunodeficiency (CVID) in young hypogammaglobulinemic children cannot yet be established despite their clinical and immunological phenotypes sharing common features with this primary immunodeficiency.

## Background

Hypogammaglobulinemia in infancy and early childhood is a heterogeneous disorder of complex pathogenesis, heterogeneous immunophenotype, a diverse clinical course and prognosis. The antibody production defect is a common feature characterized by distinct intrinsic and extrinsic factors leading to disturbed peripheral blood lymphocyte homeostasis. The developmental stages of the peripheral blood B lymph cell compartment during ontogeny encompass maturation and differentiation in an age-dependent manner, accompanied by an expression of specific immune surface and intracellular markers. This highly regulated multistep process commences in the bone marrow and is continued in the peripheral lymphoid organs, such as follicles and germinal centres of spleen and lymph nodes, as well as in the marginal zone of the spleen and underlies the composition of the peripheral blood lymphocyte compartment with distinct B cell subpopulations. The B cells outside the bone marrow are morphologically homogenous, but their surface phenotypes, anatomical localization, and functional properties are complex [1–3] and further substantial additional complexity can be revealed by a multichromatic flow cytometric approach. These circulating peripheral blood (PB) B lymphocytes can be classified according to their maturity stage into: immature/transitional, naïve, memory B cells, and plasmablasts/plasma cells. Flow cytometric immunophenotyping of peripheral blood B lymph cell compartment is a meaningful and informative tool for delineating maturation of B cell subpopulations, formation of mature B cells which enter the blood and migrate to peripheral lymphoid organs to undergo antigen-dependent activation and further differentiation to play an essential role in the adaptive immune response. The impaired B-cell development and maturational B-cell arrest and a lack of further developmental stages of B-cell lineage are associated with certain primary immunodeficiencies with hypogammaglobulinemia and lack of B-cell memory [4–8].

Profound panhypogammaglobulinemia with the presence of newly arisen B cells is observable in common variable immunodeficiency (CVID), a heterogeneous disorder in which the hallmark of the immunological phenotype is a reduction of class-switched memory B cell subset along with the accumulation of immature B cells and impaired further differentiation into plasma cells [9–11]. As the unique dynamic developmental changes in the B cell compartment are observable in infancy and early childhood, they must be taken into account when interpreting data from B cell immunophenotyping in pediatric populations [1, 12–15].

The aim of the study was to understand better the pathogenesis of hypogammaglobulinemia in infancy and early childhood based on an assessment of the peripheral blood B cell subpopulations. The evaluation of the relationship between an antibody production defect and B cell compartment will be helpful in defining the nature of the disease and its prognosis.

## Methods

### Study group

We performed a retrospective review of medical records of fifty infants and young children, 36 boys and 14 girls, aged between 3 months and 4 years (mean age 16 months, median age 14 months), who had been referred to the pediatric pneumonology, allergology and immunology university clinic because of recurrent respiratory tract infections for the purpose of differential diagnosis towards PIDs, from July through December 2014. In all the children studied hypogammaglobulinemia, below 2SD of the lower limit of age-matched reference values, regarding either exclusively IgG or combined IgG and one or two immunoglobulin isotypes, had been assessed.

### Assessment of antibody levels

The serum samples obtained from clotted peripheral blood were used for further analysis of the major classes of immunoglobulins. Immunoglobulin G, A, and M levels were measured with the use of an immunoturbidymetric assay (Beckman Coulter, USA).

### Cell preparation and peripheral blood flow cytometric immunophenotyping

Peripheral venous blood samples anticoagulated with ethylenediaminetetracetic acid (EDTA-K_2_) were stored at a temperature of between 4 and 8°C and processed within 24 hours. Cells were labelled with the following murine fluorochrome-stained monoclonal antibodies: anti-CD45 FITC (fluoresceineisothiocyanate), anti-CD14 PE (phycoerithrin), anti-CD19 PE, anti-CD19 PerCP (peridinin chlorophyll protein), anti-IgM FITC, anti-IgD FITC, anti-CD38 APC (allophycocyanin), anti-CD27 PE, and anti-CD21 FITC (Beckton-Dickinson, USA). Blood samples were mixed with antibodies, incubated in a lysing solution (FACS Lysing Solution, Beckton-Dickinson, USA), centrifuged twice and suspended in a phosphate buffered saline (PBS, Roche, Germany). The acquisition of cells and analysis were carried out with the use of the flow cytometer FACSCanto and FACSDiva software (Beckton-Dickinson, USA). With sequential gating on biparametric scattering CD45^hi^CD14^-^ lymphocytes, the following B lymph cell subpopulations were identified: memory B cells CD19^+^CD27^+^, naïve B cells CD19^+^CD27^-^, non-switched memory B cells / marginal zone B cells CD19^+^CD27^+^IgD^+^, switched memory B cells CD19^+^CD27^+^IgD^-^, transitional B cells CD19^+^CD38^hi^IgM^hi^, plasmablasts CD19^+^CD38^+^IgM^-^, and immature B cells CD19^+^CD21^lo^ and CD19^+^CD21^lo^CD38^lo^. The relative values of peripheral blood lymphocytes, the B cells of the total lymphocyte population and B cell subsets were calculated. The absolute counts of all cell subsets were calculated from the leukocyte count obtained from the analyzer XT 2000i (SYSMEX, Japan). A comparative analysis was done with a group of 111 healthy children aged from 5 months to 5 years, the peripheral blood B lymph cells immunophenotyping of whom had been carried out by Piatosa et al [14] and served to elaborate the reference cut-off values for pediatric populations at different age groups.

### Statistical analysis

The statistical analysis was conducted with the use of R 3.2.0 software. To compare sex distributions, Pearson’s chi-squared (χ^2^) tests were immediately employed. For the purpose of comparing other parameters, intervals based on median reference values in the control group (given in the study by Piatosa et al [14]) were created and subsequently, intervals in the study group were formed. To determine statistical differences between frequency distributions, a fourfold table and the χ^2^ test were applied. *P* values < 0.05 were admitted as statistically significant.

## Results

The analysis of demographic data of both the study group and the control group did not reveal statistically significant differences regarding sex and age distributions, validating the reference to age-dependent limits of a normal range, as elaborated by Piatosa et al [14].

**Fig. 1:**
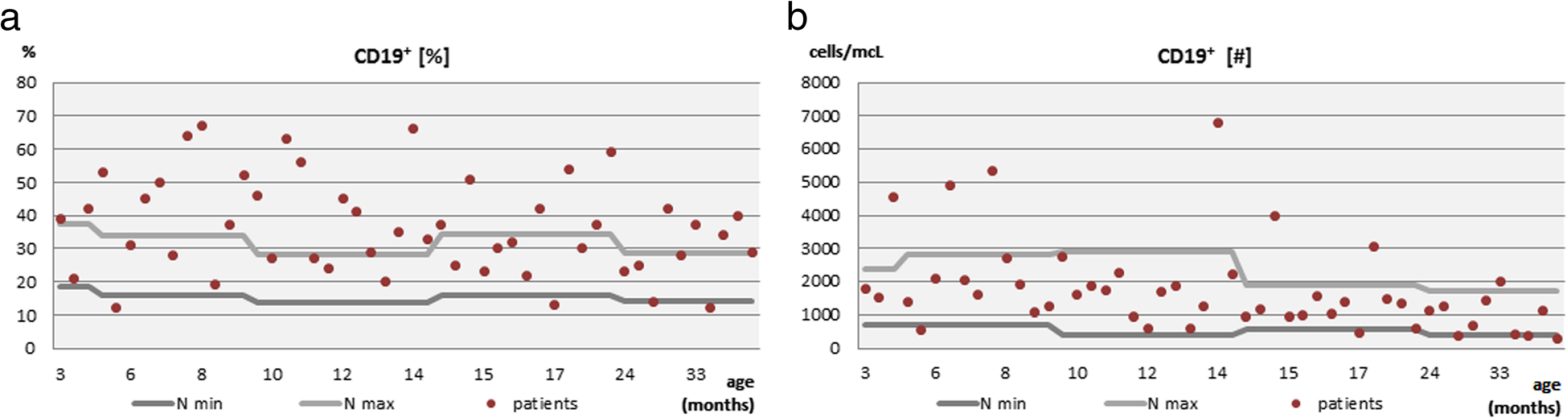
The relative frequency (**a**) and the absolute count (**b**) of the total B cell pool in hypogammaglobulinemic children

Within the total lymph cell pool, the relative frequency of the B cell population in the peripheral blood (Fig. 1a) was increased above the reference values in as much as 29 (58 %) of the children studied and it was statistically significant in children aged from 9 months to 15 months and from 15 months to 24 months (*p* = 0.002 and *p* = 0.029, respectively). At the same time it maintained within the normal range in 17 (34 %) of children and was decreased below the lower limit in only 4 (8 %) children. The tendency to increased B cell relative frequency was accompanied by a normal absolute B cell count (Fig. 1b), which was assessed in as many as 37 (74 %) of the children studied.

**Fig. 2:**
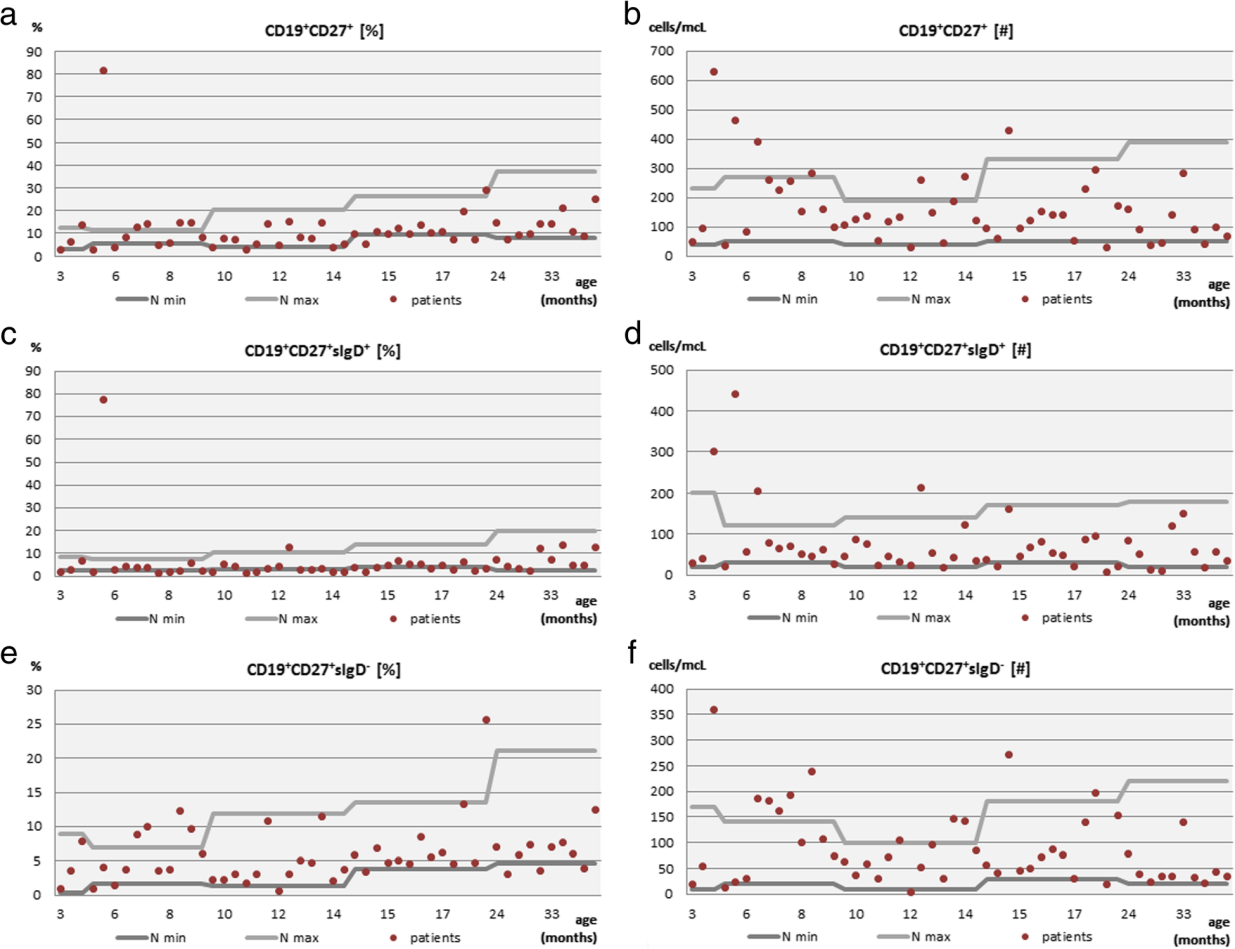
The immunophenotypic distribution of memory B cells in the children studied: the relative frequency (**a**) and the absolute count (**b**) of the total memory B cell pool, non-switched memory B cells (**c** and **d**) and the switched memory B cell subpopulation (**e** and **f**)

The total memory B cell subset was within age-matched limits both in terms of relative value and in terms of an absolute count in 32 (64 %) and 37 (74 %) of the children studied, respectively (Fig. 2a and 2b). A decrease in the absolute count of the total memory B cell population occurred admittedly in only 6 (12 %), but in groups of children aged from 15 to 24 months and from 24 to 60 months the decrease of a memory B cell number was statistically significant (*p* = 0.029 and *p* = 0.024, respectively). The relative memory B cell frequency was decreased in 11 (22 %) of the children studied and in children aged from 24 to 60 months it was statistically significant (*p* = 0.0245). The impaired B cell memory was accompanied by an increase both in the relative frequency and absolute number of naïve B cells in 7 (14 %) of the children. The increase of the relative frequency of naïve B cells was observable in three age groups: in children from 9 to 15 months, from 15 to 24 months as well as from 24 to 60 months of age and it was statistically significant (*p* values 0.043, 0.029 and 0.0245, respectively).

Within the memory B cell pool, non-switched (marginal zone-like, MZL) and switched memory cells were distinguished. The relative frequency of MZL B cells (Fig. 2c) was decreased in total in as many as 22 (44 %) children, whereas in children aged from 9 to 15 months and in children aged from 15 to 24 months the decrease of MZL B cells compared to the control group was statistically significant (*p* values 0.012 and 0.001, respectively). The absolute count of MZL B cells was decreased in 10 (20 %) of the children studied and again, in the group of older patients, from 15 to 24 months of age, the decrease of the MZL B cell subset was statistically significant (*p* = 0.007).

The switched memory B cell subset in general maintained within the normal age-matched range in terms of both relative value and absolute count (Fig. 2e and 2f, respectively) in 38 (76 %) patients. However, the decrease of relative values and absolute numbers of this B cell subset that was observed in total in 12 (24 %) patients, was statistically significant in the older age group, namely in children aged from 9 to 15 months and from 24 to 60 months (*p* = 0.043 and *p* = 0.025, respectively).

**Fig. 3:**
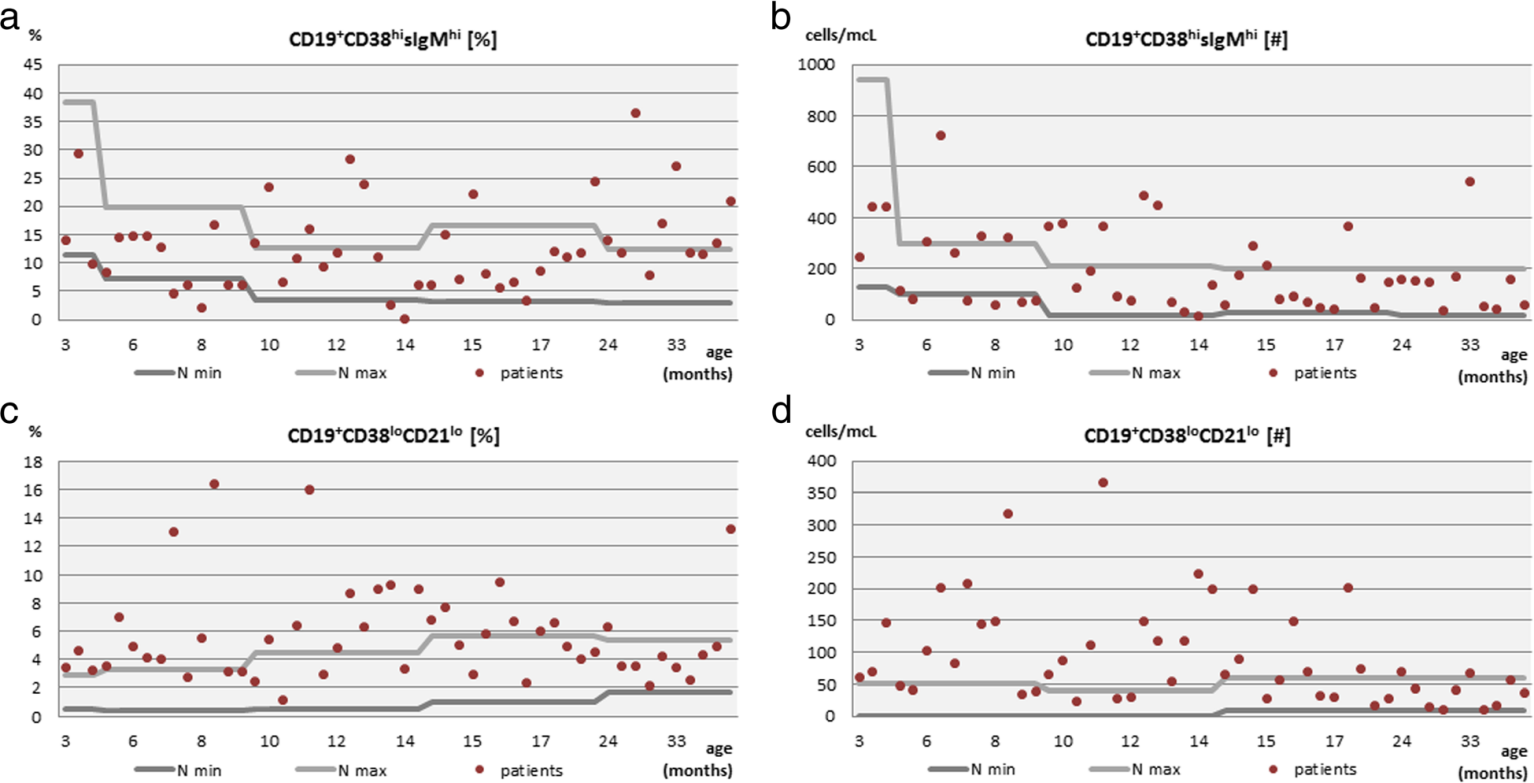
The relative frequency and the absolute count of transitional B cells (**a** and **b**) and immature B cells with low expression of CD21 (**c** and **d**)

A transitional B cell subset, in turn, showed a tendency to increase its relative frequency (Fig. 3a) in 10 (20 %) and the absolute count (Fig. 3b) in 12 (24 %) the children studied and again, the increase of the relative frequency of transitional B cells was statistically significant (*p* = 0.004) in children aged from 24 to 60 months. The absolute count as well as the relative frequency of transitional B cells maintained normal in 30 (60 %) and 29 (58 %) of patients, respectively.

The percentage of the immature B cell pool, defined as CD19^+^CD21^lo^, was below the age-matched cut-off values in 10 (20 %) of the children studied and of these, 9 were children aged less than 18 months. The immature B cell subset was further specified by their expression of CD38. The increased percentage and the absolute count above the age-matched reference values of the CD19^+^CD21^lo^CD38^lo^ B cell subset was apparent in 29 (58 %) of the children studied (Fig. 3c and 3d, respectively). However, it is worth noting that the expansion of these more immature B cells was observable in as much as 26 of children aged from 3 to 17 months, but it was statistically significant in the group of patients aged between 24 and 60 months (*p* = 0.004).

Interestingly, the number and the relative frequency of plasmablasts was increased above the reference values in 13 (26 %) patients and maintained within the normal range in 31 (62 %) and 29 (58 %) of all our hypogammaglobulinemic children, respectively. However, a distribution of the absolute count of plasmablasts reaching both the lower or higher limit of the normal range can be noticed, but the analysis did not reveal the statistical significance in any age group of the children studied.

## Discussion

Diagnostic criteria of the vast majority of primary immunodeficiencies with antibody production defects are based on anamnesis which includes family history and individual medical history, a clinical manifestation of the disease and the exclusion of other diagnoses causing secondary hypogammaglobulinemia. Antibody production defects are the most common and the largest group of primary immunodeficiency disorders in children, with heterogeneity of the clinical course and prognosis, ranging from mild or even asymptomatic selective IgA deficiency or transient hypogammaglobulinemia of infancy (THI) to very severe agammglobulinemias with the profound inability to mount the antigen-specific humoral immune response [16]. Peripheral blood lymph cell immunotyping with assessment of B cell pool maturation is an important step in the differential diagnosis of symptomatic hypogammaglobulinemia and particularly in common variable immunodeficiency (CVID) abnormalities in B cell subsets have been identified [17, 18]. Depending on the relative frequency of peripheral blood class-switched memory B cells, Freiburg [19], Paris [20] and EUROclass [9] classification schemes have been proposed in order to find the correlation between B lymph cell maturational defects and clinical manifestation and to identify patients with a high risk of specific complications, such as autoimmunity, splenomegaly and granulomatous disease. However, taking into consideration the maturational development of the immune competence in children, with the most dynamic shifts among lymph cell subsets in peripheral blood reflecting structural and functional maturation of central and peripheral immune organs, the predictive role of class-switched memory B cells in pediatric patients with antibody production defects may be called into question [21].

In our study the hypogammaglobulinemic children manifested diverse maturational defects within the B lymph cell pool. Firstly, in a large group of them, an increase of the total B cell relative frequency was observable. Interestingly, it was statistically significant in children between 9 and 24 months, but not in young infants between 2 and 9 month of age. Similarly, Dorsey and Orange reported an increase in the B cell percentage in a group of 24 children with IgG hypogammaglobulinemia and / or decreased other immunoglobulin izotypes [22]. The authors suggested the increased B lymph cell proportion was a characteristic feature of transient hypogammaglobulinemia of infancy and concluded that THI is associated with different abnormalities regarding B cell-related immune response beyond just hypogammaglobulinemia. While the number and relative frequency of the total B lymph cell pool is not widely considered in children as an exclusive predictive factor of possible development of CVID in hypogammaglobulinemic children, Aydogan et al [23] nevertheless considered a decrease in the percentage of the B cell number as an important immunologic parameter for identifying pediatric CVID patients at risk of respiratory complications. In the studies by Piatosa et al [24] and by Bukowska-Strakova et al [25] the authors showed a decrease in the total number of B cells in a proportion of pediatric CVID patients. As in the latter study, THI children had normal number and relative frequency of total B cells, so the authors concluded that hypogammaglobulinemia in THI results rather from inappropriate immunoregulation than with disturbance in development of the B cell compartment.

Considering the clinical heterogeneity of antibody deficiencies and peculiar obstacles in establishing a definitive diagnosis in children aged less than 5 years, a detailed immunophenotypic examination of the B-cell subpopulations with the use of age-specific cut-off reference values is of critical importance, but none of the above mentioned classification schemes clearly defines such age-matched values. Whereas many authors admittedly recommend an analysis of the memory B cell population to facilitate the diagnosis of CVID in pediatric patients [25–30], Smet et al [21] showed that the EUROclass limit of 2 % of B cells being switched memory [9] as well as the Freiburg limit of 0.4% of switched memory B cells among total peripheral blood B cells [19] were already reached by healthy children from 1 year of age onwards. Nonetheless, the authors pointed out the cut-off value of 8 % of switched memory B cells obtained in an adult cohort by the French group [20] was not suitable for healthy children even between the ages of 10 and 15 years, but their limit value of 11% for total memory B cells appeared to be applicable from the age of 2 years upwards.

In our study group, in 10 children aged 24 month or more, 6 (60 %) of them had a total memory B cell percentage lower than 11 and in this age group the memory B cell deficiency was statistically significant. Compared to the control group, the memory B cell frequency was below the age-matched limit in 11 (22 %) of all hypogammaglobulinemic children studied in general, but in children younger than 15 month of age it was not statistically significant.

The switched memory B cell subpopulation reached the limit of 0.4 % among total B cell pool in all our children studied and the relative value of switched memory cells was below 2 % in 5 patients, aged from 3 to 12 months, but not in any children studied from the age of 1 year upwards. As was stressed by Smet et al [21], the particular limits of B cell subpopulations admitted as a diagnostic criteria by EUROclass and Freiburg classifications may be applicable even for young children, but a French classification has more limitations if applied to children.

As was shown by Bukowska-Strakova et al [25] and Karaca et al [31], patients with THI do not show substantial abnormalities regarding the peripheral blood B cell pool and the percentage of memory B cells remains within the age-matched limits. Similarly, in a group of children with the initial diagnosis of THI reported by Moschese et al [32], most of these patients recovered from hypogammaglobulinemia by the age of 24 months and did not show a deficiency in switched memory B cells. These children, who did not normalize their immunoglobulin levels by the age of 24 months, expressed a reduced frequency of switched memory B cell pool and shared clinical and immunological profiles similar to other primary immune deficiencies with antibody production defects. In the children studied, we did not find a significant correlation between the severity of hypogammaglobulinemia, in particular IgG deficiency, and defective development of any B cell population.

In a proportion of CVID patients, an observable B cell maturation defect with reduced switched memory B cell development is also associated with expansion of transitional CD19^+^CD38^hi^IgM^hi^ and CD21 positive B cells or more immature B cell subset with low expression of CD21 maturation marker [33, 34], which correlated with clinical phenotypes such as lymphadenopathy and splenomegaly as well as autoimmune cytopenias [35]. Importantly, the abnormally high percentage of these CD21^lo^ transitional B cells in circulation which was shown in infants in our study group may be observed during the first years of life and is attributable to immaturity of the immune system in the early childhood [36–39]. As the evolution of percentages of CD21^lo^ B cells is age-related, application of EUROclass criteria regarding this immature B cell subset for the diagnosis of B cell dysfunction requires careful standardization of flow cytometry evaluation of B cell maturation and for the purpose to its use as a prognostic marker of immunodeficiency in children.

Certainly, a lack of the follow-up of the study group that could add more significance to the immunological data is the major drawback of the presented study. However, assessment of the B cell subsets of our children can be considered as a screening evaluation for the purpose to identifying high-risk patients who require regular follow-up visits and further evaluation of their immune system.

## Conclusions

The greatest and the most dynamic developmental and maturational changes among B cell subsets occur in children below the age of 2 years. The immaturity of the immune system in the infancy and early childhood contribute to the age-related B lymphocyte abnormalities. Therefore, the reliable diagnosis of CVID in hypogammaglobulinemic children aged less than 2 years cannot yet be established despite their clinical and immunological phenotypes sharing common features with this disease entity. It needs to be highlighted that the classifications used in adults cannot be applied to children with antibody production defects who therefore require separate diagnostic and prognostic criteria.

## Abbreviations

APC, allophycocyanin; CD, cluster of differentiation; CVID, common variable immunodeficiency; EDTA, ethylenediaminetetracetic acid; FITC, fluoresceineisothiocyanate; PB, peripheral blood; PE, phycoerithrin; PerCP, peridinin chlorophyll protein; PID, primary immunodeficiency; MZL, marginal zone-like; THI, transient hypogammaglobulinemia of infancy
